# α1-Blockers and 5α-Reductase Inhibitors Are the Most Recommended Drugs in Treating Benign Prostatic Hyperplasia: An Evidence-Based Evaluation of Clinical Practice Guidelines

**DOI:** 10.3389/fphar.2020.00311

**Published:** 2020-03-25

**Authors:** Xiao-Feng Xu, Guo-Xiong Liu, Cong Zhu, Xi-Min Qiao, Shao-Fu Yu, Tong Deng, Ying-Hui Jin

**Affiliations:** ^1^Department of Urology, Xianyang Central Hospital, Xianyang, China; ^2^Center for Evidence-Based and Translational Medicine, Zhongnan Hospital of Wuhan University, Wuhan, China; ^3^Department of Urology, Zhongnan Hospital of Wuhan University, Wuhan, China; ^4^Department of Pharmacy, The Huaihua Second People's Hospital, Huaihua, China; ^5^Center for Evidence-Based Medicine, Institute of Evidence-Based Medicine and Knowledge Translation, Henan University, Kaifeng, China

**Keywords:** clinical practice guideline, benign prostatic hyperplasia, evidence-based evaluation, AGREE II instrument, medical treatment

## Abstract

**Objective:**

To systematically evaluate the quality of clinical practice guidelines (CPG) for medically treating benign prostatic hyperplasia (BPH), and to compare the context of recommendations in order to provide references for clinical application.

**Methods:**

We searched databases of National Guideline Clearinghouse (NGC), Guidelines International Network (GIN), National Institute for Health and Clinical Excellence (NICE), Scottish Intercollegiate Guidelines Network (SIGN) and World Health Organization (WHO), PubMed, Embase, CNKI, VIP, WanFang Data, CBM, and Medlive from their establishment to October 13, 2019, to collect evidence-based guidelines and/or consensus on BPH. Method quality of included guidelines was assessed according to the Appraisal of Guidelines for Research and Evaluation II (AGREE II) instrument, and differences and similarities among recommendations were compared.

**Results:**

A total of 22 guidelines were included, of which eight were updated versions. According to the AGREE II instrument, the median score of scope and purpose, stakeholder involvement, rigor of formulate, clarity of presentation, applicability, and editorial independence was 71.5%, 41%, 25%, 64%, 18%, and 28%, respectively. Based on recommendations for medical treatment, almost all guidelines recommended α1-blockers and 5α-reductase inhibitors, and most guidelines also recommended muscarinic receptor antagonists. In terms of drug combination therapy, most guidelines recommended “α1 blockers and 5α-reductase inhibitors”, and some guidelines also recommended “α1 blockers and muscarinic receptor antagonists”.

**Conclusion:**

The recommendations from different guidelines were basically similar, only showing conflicts in some areas. The quality of included guidelines remains to be unified, and their context can provide valuable implications for development or improvement.

## Introduction

A meta-analysis on studies from 25 countries showed that the lifetime prevalence of BPH was 26.2% [95% confidence interval (CI): 22.8–29.6%] and there were no regional or ethnic differences (Lee et al., 2017). In addition, in the United States alone, the annual spending on BPH treatment is estimated to be approximately $4 billion (Taub and Wei, 2006). With the advent of an aging society, BPH has become a serious burden to clinical work, society, and economy. The development and continuous updating of the BPH Clinical Practice Guide (CPG) (Wang, 2016) impose a positive impact on promoting the standardization of clinical medical work. In recent years, many countries, especially developed ones, have made great achievements in the development and application of BPH diagnosis and treatment guidelines in order to solve many problems faced in BPH clinical practice (Novara et al., 2006). Despite this progress, the quality of many CPG still appeared to fall below desirable standards. Therefore, this article studied and analyzed the basic content and development trend of global BPH clinical guidelines, used the AGREE II tool to scientifically evaluate the guidelines, compared the advantages and disadvantages of each guide from six domains. And focused on the content of drug treatment for BPH guidelines, hoping to provide help for frontline clinicians when referring to the guidelines, and also hoping to provide references for the specification of evidence-based guidelines for clinical treatment.

## Methods

### Inclusion and Exclusion Criteria

Inclusion globally published BPH-field clinical practice guidelines or consensus (the latest version) that meets the guidelines and is developed and issued by academic or national authorities. Guidelines must include recommendations for drug therapy. Exclude foreign direct translations or adapted foreign guides, guide interpretation documents, technical or operational instructions, lectures or expert writing, and knowledge manuals.

### Search Strategy

Computer searched National Library of the United States (NGC), Guideline International Network (GIN), National Institute of Health and Clinical Demonstration (NICE), English Inter-Institutional Guide Network (SIGN), World Health Organization (WHO), PubMed, Embase, China National Knowledge Infrastructure (CNKI), Wanfang database, VIP database, China Biomedical Literature Data Road, and Medlive website from their inception to October 20, 2019, and a manual retrieval was also performed for relevant literature references. No language restrictions were applied to the search strategies. The search terms included BPH, benign prostate hyperplasia, enlarged prostate, BPH, prostatomegaly, prostatauxe, prostatic hypertrophy, benign prostatic enlargement, benign prostatic obstruction, lower urinary tract symptoms, LUTS, guideline, specification, etc.

### Literature Screening and Data Extraction

The two evaluators independently completed literature screening and cross-checking according to the inclusion and exclusion criteria. If there were objections, the third evaluator would participate in the discussion and resolve the differences. Data were extracted according to a pre-designed data extraction table, and the extracted contents included the names of guideline, releasing country and organization, the earliest release or updating time, research area, drug treatment opinions, formulation methods, and references.

### Quality Assessment

Pre-scoring was performed three times before formal scoring, and consistency was tested using intra-group correlation coefficient (ICC). ICC is one of the reliability coefficient indicators for measuring and evaluating the reliability between observers and retest reliability. Then, methodological quality was evaluated by two reviewers using the AGREE II (Wang, 2016) (**Supplementary Table 1**). The AGREE II consists of 23 key items organized within six domains followed by two global rating items (“overall assessment”). The six domains are “scope and purpose”, “stakeholder Involvement”, “rigor of development”, “clarity of presentation”, “applicability”, “editorial independence”. The two assessors received education regarding to the guideline development process and evidence-based nursing and were trained on the use of AGREE II. After the evaluation, answers from them were compared, and the score difference for each item greater than two points was defined as a large difference. Then, the two reviewers would give a new score after discussion. If differences still existed, a professor with extensive experience in using AGREE II would help. Three reviewers would combine all supporting materials and opinions to arrive a final score. We analyze the quality of included guidelines according to the following scheme: (1) score 23 key items within six domains; (2) each of the AGREE II items are rated on a seven-point scale (1–strongly disagree to 7–strongly agree); (3) the consistency of evaluations between the two reviewers was judged through calculating ICC value; score: domain score = (actual score-minimum possible score)/(maximum possible score-minimum possible score) × 100%. The higher the domain standardization score, the more complete the method, and the reporting when guidelines for the domain were developed.

### Statistical Analysis

Descriptive analysis and presentation of results were completed using Excel 2007 software, and ICC values were calculated using SPSS 19.0 software. ICC value ranged between 0 and 1, and consistency would be poor when ICC value was less than 0.4. As for ICC locating between 0.4 and 0.75, it meant that the consistency was average. When ICC ≥ 0.75, the consistency was fine. ICC value should be above 0.7.

## Results

### Basic Features of Literature Search Results and Guidelines

**Figure 1:**
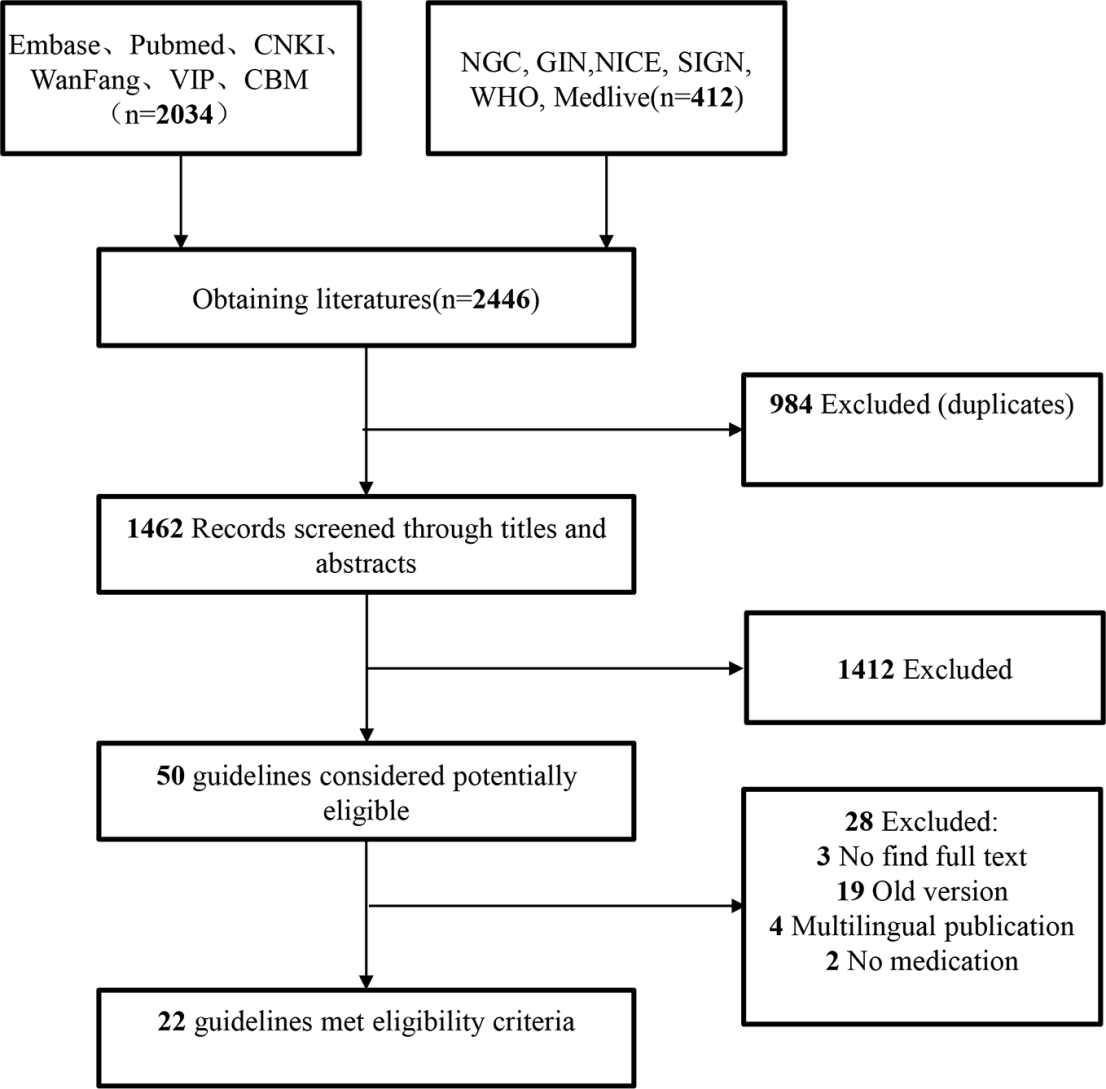
Literature search and screening process.

A total of 2,562 articles were obtained in the preliminary searching. After layer-by-layer screening, 22 guidelines were finally included (Cockett et al., 1991; Chang, 1998; Bereczky et al., 2006; Cavalcanti et al., 2006; Hofner, 2007; Zhang et al., 2007; Mcvary et al., 2011; Zhu et al., 2011; Spatafora et al., 2012; Tammela et al., 2012; Chapple, 2015; Gratzke et al., 2015; Wang, 2015; Yeo et al., 2016; Zhang et al., 2016; Homma et al., 2017; Sun et al., 2017; Yu and Gao, 2017; Zhang et al., 2017; Geng, 2018; Gravas et al., 2019; Nickel et al., 2018). The document screening process and results are shown in **Figure 1**. Of the 22 included guidelines, 2 were from European urology association (Gratzke et al., 2015; Gravas et al., 2019), eight from China (Zhang et al., 2007; Zhu et al., 2011; Wang, 2015; Zhang et al., 2016; Sun et al., 2017; Yu and Gao, 2017; Zhang et al., 2017; Geng, 2018), while only one from Japan (Homma et al., 2017), Brazil (Cavalcanti et al., 2006), Finland (Tammela et al., 2012), Germany (Hofner, 2007), the United Kingdom (Chapple, 2015), WHO (Cockett et al., 1991), Italy (Spatafora et al., 2012), Malaysia (Chang, 1998), Canada (Nickel et al., 2018), the United States (Mcvary et al., 2011), South Africa (Bereczky et al., 2006), and Korea (Yeo et al., 2016), respectively. The basic characteristics of the included guidelines are shown in **Table 1**.

**Table 1:** Information about the inclusion guide.

Inclusion guideline	Country/International organization	Publish/Last update time	Guideline name	Publishing organization	Field
Gratzke et al., 2015	Europe	1998/2015	EAU Guidelines on the Assessment of Non-neurogenic Male Lower Urinary Tract Symptoms including Benign Prostatic Obstruction	European Association of Urology (EAU)	Diagnosis and Treatment
Zhang et al., 2007	China	2007	Guideline for clinical diagnosis and treatment of benign prostatic hyperplasia	Chinese Medical Association Urology Branch	Diagnosis and Treatment
Homma et al., 2017	Japan	1999/2011/2017	Clinical guidelines for male lower urinary tract symptoms and benign prostatic hyperplasia	Japanese Society of Urology	Diagnosis and Treatment
Zhu et al., 2011	China	2011	Elderly patients with benign prostatic hyperplasia/lower urinary tract symptoms drug treatment consensus	Chinese Medical Association Geriatrics Branch	Medical Treatment
Cavalcanti et al., 2006	Brazil	2006	Benign prostatic hyperplasia	Brazilian Urological Association/Brazilian Medical Association	Diagnosis and Treatment
Tammela et al., 2012	Finland	2006/2012	Benign prostatic hyperplasia	Finnish Medical Society Duodecim	Diagnosis and Treatment
Höfner, 2007	Germany	2007	Treatment of Benign Prostate Syndrome (BPS)	German Society of Urology (DGU) and Professional Association of German Urologists (BDU)	Treatment
Chapple, 2015	United Kingdom	2010/2015	Lower urinary tract symptoms in men: management	NICE	Diagnosis and Treatment
Cockett, et al., 1991	WHO	1991	World Health Organization Consensus Committee recommendations concerning the diagnosis of BPH	WHO	Diagnosis and Treatment
Spatafora et al., 2012	Italy	2007/2012	Evidence-based guidelines for the treatment of lower urinary tract symptoms related to uncomplicated benign prostatic hyperplasia in Italy: updated summary from AURO.it	Italian Association of Urologists	Treatment
Chang, 1998	Malaysia	1998	Consensus on Management of Benign Prostatic Hyperplasia	Malaysian Urological Association and Prostate Health Council of Malaysia	Diagnosis and Treatment
Nickel et al., 2018	Canada	2005/2010/2018	Canadian Urological Association guideline on male lower urinary tract symptoms/benign prostatic hyperplasia (MLUTS/BPH): 2018 update.	Canadian Prostate Health Council and the CUA Guidelines Committee	Diagnosis and Treatment
McVary et al., 2011	United States	1994/2011	Update on AUA guideline on the management of benign prostatic hyperplasia	American Urological Association (AUA)	Diagnosis and Treatment
Bereczky et al., 2006	South Africa	2006	Management of benign prostatic hyperplasia - South African Urological Guideline	South African Urological Association	Diagnosis and Treatment
Wang, 2015	China	2015	Elderly patients with benign prostatic hyperplasia/lower urinary tract symptoms drug treatment consensus	Chinese Medical Association Geriatrics Branch	Medical Treatment
Yeo et al., 2016	Korea	2016	Korean clinical practice guideline for benign prostatic hyperplasia	The Korean Urological Association	Diagnosis and Treatment
Zhang et al., 2016	China	2016	Expert consensus on Chinese Medicine diagnosis and treatment of benign prostatic hyperplasia	China Association of Chinese Medicine, Men's Branch	Diagnosis and Treatment
Sun et al., 2017	China	2017	Guidelines for the diagnosis and treatment of benign prostatic hyperplasia with integrated traditional Chinese and Western medicine (Trial version)	Chinese Association of integrative Medicine, Men's Branch	Diagnosis and Treatment
Yu and Gao, 2017	China	2017	Clinical application of Ningbitai Capsule in the treatment of lower urinary tract symptoms	China Information Association of Traditional Chinese Medicine, Men's Branch	Medical Treatment
Zhang et al., 2017	China	2017	Expert consensus on treating benign prostatic hyperplasia based on kidney deficiency and phlegm	China Association of Chinese Medicine, Men's Branch	Diagnosis and Treatment
Geng, 2018	China	2018	Chinese expert consensus on the clinical application of Huangqi Capsule in benign prostatic hyperplasia	China Information Association of Traditional Chinese Medicine, Men's Branch	Medical Treatment
Gravas et al., 2019	Europe	2011/2018/2019	Management of Non-neurogenic Male LUTS	European Association of Urology (EAU)	Diagnosis and Treatment

### AGREE II Evaluation Results

The results of consistency test showed that the ICC values of all guidelines were > 0.735 (0.735 to 0.994), indicating that their consistency was fine. The results of standardized scores in the six domains are shown in **Table 2**.

**Table 2:** Results of AGREE II evaluation.

Inclusion of guidelines	Standardized scores in various domains (%)					
	Scope and purpose	Stakeholder Involvement	Rigor of Development	Clarity of Presentation	Applicability	Editorial Independence
Gratzke et al., 2015	82	60	71	78	47	92
Zhang et al., 2007	71	36	30	58	16	79
Homma et al., 2017	80	36	56	80	19	38
Zhu et al., 2011	63	29	10	49	7	0
Cavalcanti et al., 2006	72	44	30	46	7	60
Tammela et al., 2012	65	29	16	53	0	23
Hofner, 2007	74	49	59	72	29	67
Chapple, 2015	74	68	60	76	51	58
Cockett et al., 1991	61	38	16	50	16	19
Spatafora et al., 2012	76	38	50	78	25	31
Chang, 1998	60	24	10	47	8	4
Nickel et al., 2018	81	64	76	83	46	79
Mcvary et al., 2011	78	49	53	79	48	85
Bereczky et al., 2006	51	24	4	33	11	25
Wang, 2015	78	47	20	64	19	0
Yeo et al., 2016	70	36	51	64	11	34
Zhang et al., 2016	78	56	14	80	2	0
Sun et al., 2017	67	47	14	36	17	0
Yu and Gao, 2017	61	44	4	44	23	0
Zhang et al., 2017	67	30	14	38	11	0
Geng, 2018	70	30	17	78	38	0
Gravas et al., 2019	86	56	69	83	63	83
Median	71.5	41	25	64	18	28
Q1	64	30	14	46.5	9.5	0
Q3	78	52.5	57.5	78.5	42	73
Range	35	24	72	50	63	92

Q1, 1st quartile; Q3, 3rd quartile.

#### Scope and Purpose

The median (Q1, Q3) and full range in the domain were 71.5% (64%,78%) and 35%. The median score was highest in all areas. Almost all guides were well in this area, and no guideline score was below 50%.

#### Stakeholder Involvement

The median (Q1, Q3) and full range in this area were 41% (30%, 52.5%) and 24%. The median score ranked the third position across all areas, and the full range score was higher than the median score. The minimum score in this area was 22%, and only four guides possessed scores greater than 50%.

#### Rigor of Development

The median (Q1, Q3) and full range in the domain was 25% (14%, 57.5%) and 72%. The median score ranked the third position among the six areas, and the full-range score was much larger than the median score. Five guides in the domain exhibited a median score greater than 50% and two guides showed a minimum score of 4%.

#### Clarity of Presentation

The median (Q1, Q3) and full range score in this area was 64% (46.5%, 78.5%) and 50%. The median score ranked the second position in all areas. Most guides were well in this area, and only six guides displayed scores below 50%.

#### Applicability

The median score in this domain was lowest across all domains (18%). Only one guideline scored above 50%, while one scored 0.

#### Editorial Independence

The median (Q1, Q3) and full range (full range) score for this domain were 28% (0,73%) and 92%. In this area, five guides scored over 70%, and 7 scored 0.

### Medication Recommendations

**Figure 2:**
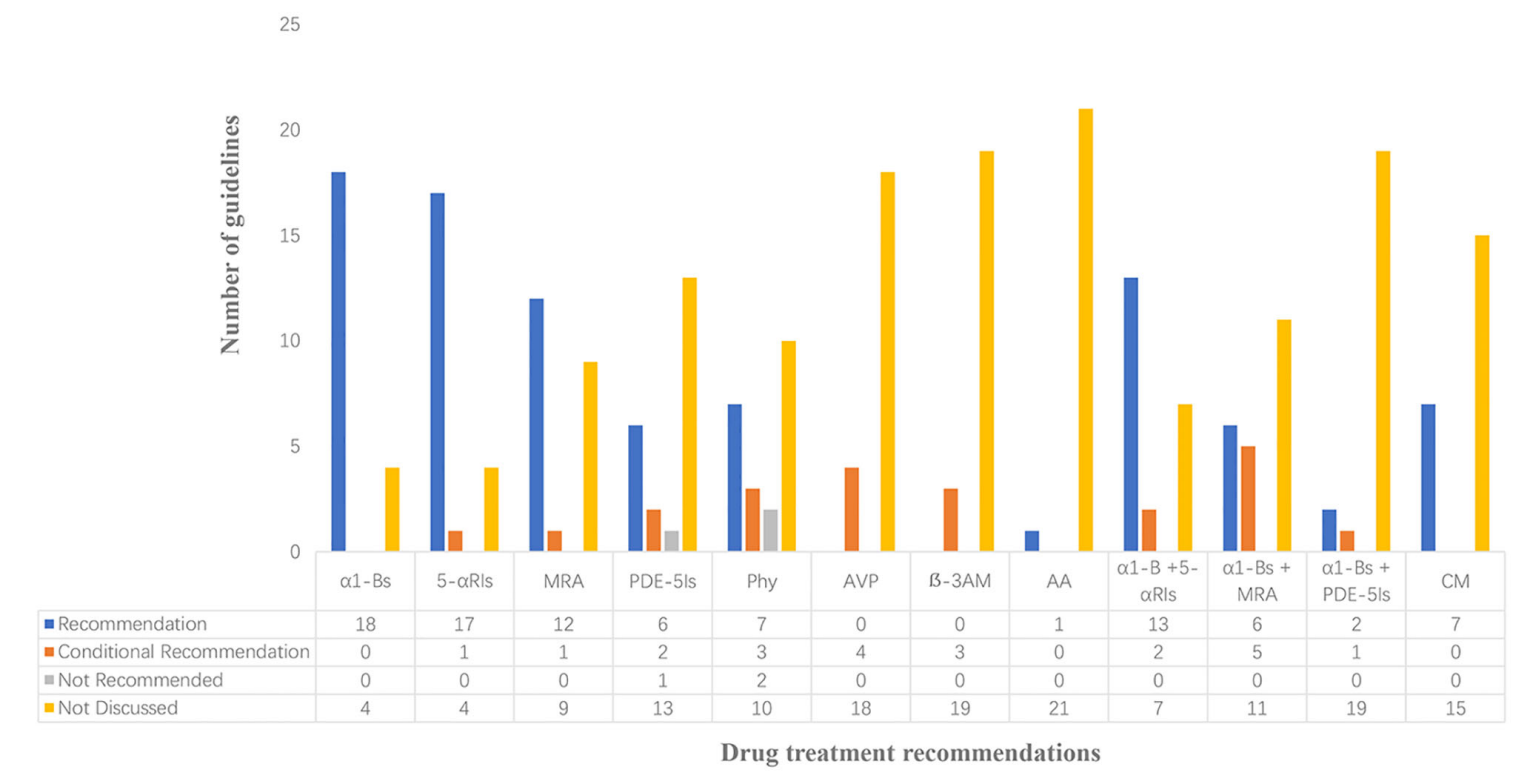
Analysis of drug treatment opinions α1-Bs, α1-Blockers; 5-αRIs,5α-Reductase inhibitors; MRA, Muscarinic receptor antagonist; PDE-5Is, Phosphodiesterase 5 inhibitor; Phy, Phytotherapies; AVP, Arginine vasopressin; β-3AM, Beta-3 agonist medications; AA, Anti-androgen; α1-B+5-αRIs, α1-Blockers+5α-Reductase inhibitor; α1-B+MRA, α1-Blockers + Muscarinic receptor antagonist; α1-B+PDE-5Is, α1-Blockers + phosphodiesterase 5 inhibitor; CM, Chinese medicine.

Guidelines for drug treatment recommendations were shown in **Figure 2**. Four of the included guidelines purely involved traditional Chinese medicine, including no recommendations for other treatments. Almost all of the guidelines recommended α1-blockers and 5α-reductase inhibitors, and most of the guidelines recommended muscarinic receptor antagonists. Meanwhile, eight guidelines recommended the use of phosphodiesterase 5 inhibitor, while one guideline from China considered that currently, phosphodiesterase 5 inhibitor in our country had no indications for BPH/LUTS treatment, so such drug was not recommended for the time being. There were 10 guidelines recommending phytotherapy, but there were also two guidelines not recommending such approach for BPH. Although phytotherapy is popular in many parts of the world, both European and South African guidelines believed that currently, there was no objective evidence confirming its efficacy, mode of action, or biological effect. Besides, four guidelines recommended arginine vasopressin which was mainly adopted for treating polyuria at night. Three guidelines addressed recommendations for beta-3 agonist. Only seven guidelines from China and Japan recommended Chinese medicine treatment. Japanese guidelines recommended anti-androgen therapy alone. Guidelines for combined medication had a high degree of uniformity in recommending “α1-blockers and 5α-reductase inhibitors”, and 11 guidelines recommended “α1-blockers and muscarinic receptor antagonists”. A total of three guidelines from Japan, Germany, and China, respectively, recommended a combination of “α1-blockers and phosphodiesterase 5 inhibitors”.

## Discussion

This study aimed to evaluate the quality of CPG for BPH worldwide. We identified 22 CPGs related to BPH, which were published between 1991 and 2019. The median and range scores for the six AGREE II domains (scope and purpose, stakeholder involvement, rigor of development, clarity of presentation, applicability, and editorial independence) were 71.5%, 41%, 25%, 64%, 18%, and 28%. An increasing number of CPGs are being published. However, there are considerable potentials to elevate the quality of each domain. In the six major domains of the AGREE II tool scoring system, scores only in domains 1, “scope and purpose” and 4 “clarity of presentation” were >50%. Therefore, scores in the other four areas need to be improved. The median (range) of the scope and purpose scores was 71.5% (35%), indicating that these guidelines clearly described their ranges and purposes, and could help users to quickly determine whether they were what you needed. The stakeholder participation rate was 41% (24%), mainly because most guidelines did not take into account the views or wishes of target populations (patients, the public, etc.) of item 5. The rigor of development was 25% (72%), with a lower median and a larger range, indicating that a few criteria met the standards in the domain, and most guidelines did not report systematic retrieval or recommendation formation methods in the article. The clarity of the report was 64% (50%), indicating that the included guidelines met the criteria for most projects in the domain, but the recommendations of some guidelines were vague and difficult to identify quickly. The South African guidelines with the lowest scores (33%) recommended treatment but did not provide indicators such as duration and dose. The applicability was 18% (63%), mainly because the guidelines offered ambiguous descriptions on facilitators and obstacles during application, and only a small number of them provided different versions and supporting documents. Almost all guidelines did not mention potential resource inputs. The editorial independence was 28% (92%), the median was low, but the range was large, and only a few guidelines not only provided funding units but also clearly indicated whether they were affected by funding agency. Most guidelines either did not report funding agencies or reported funding agencies but did not state conflicts of interest.

In terms of drug treatment recommendations, they were basically the same. BPH is mainly featured by histological prostatic hyperplasia and glandular components, anatomical enlarged prostate (BPE), urodynamic bladder outlet obstruction (BOO), and low urinary tract symptoms (LUTS) (Wang, 2014). In treating BPH, pharmacological therapy may be not as effective as surgical therapy, but could sufficiently relieve symptoms for many patients, causing fewer adverse events (Kirby, 2000). Studies have shown that about 85% of patients receiving conservative treatment enjoy stable statuses during a follow-up of 1 year, and about 65% show no clinical progress within 5 years (Wang, 2014). Therefore, almost all guidelines reached a relatively uniform opinion on conservative treatment options. α1-Blockers, 5α-reductase inhibitors, and muscarinic receptor antagonists were recommended by most guidelines. Some research results suggested that the efficacy of plant preparations in BPH was equivalent to that of blockers and 5a proenzyme inhibitors, and that there were no obvious adverse reactions for plant preparations (Fourcade et al., 2008). Therefore, it has been recommended by Germany, China, Japan, Brazil, Finland, and Canada guidelines. Drugs such as phosphodiesterase 5 inhibitor, arginine vasopressin, and beta-3 agonist medications were also recommended by some guidelines, but such recommendation was not replicated by most guidelines. Regarding to the use of Chinese medicine in BPH, except those from China and Japan, guidelines from other countries did not discuss this aspect.

Our review had several strengths. First, we attempted to cover all published guidelines for our systematic review of qualities of CPGs on BPH. Our structured and explicit approach increased the validity of the findings. Second, we used the AGREE II instrument, which is a scientific and valid tool to assess the quality of CPGs. There were also some limitations in this study. First of all, the tool AGREE II, when targeting guidelines, only focused on evaluating their development methodology and the quality of their reporting. Consequently, the evaluation on their evidence quality and the authenticity of their recommendations was not enough. And score might fail to truly reflect quality. In addition, although evaluation scores are helpful in comparing the quality of clinical guidelines, they could do little in elevating the quality. Second, we did not limit expert consensus, and it differed from clinical practice guidelines in format and production methods. This was also a possible reason for lower AGREE II score in some areas. Finally, this study showed restriction in language, and clinical guidelines published in other databases might also be missed.

## Conclusion

In summary, the overall quality of the included guidelines is uneven and needs to be unified. According to our analysis on the recommended uniformity of the acceptance guidelines, it could be concluded that in terms of drug treatment, α1-blockers and 5α-reductase inhibitors are more mature drugs for BPH treatment; therefore, it is recommended that in the future when CPG is formulated/revised Ability to use recognized standards wherever possible. Of course, in the light of actual conditions in countries and regions, based on recognized standards, it is also allowed to modify them to suit corresponding standards.

## Data Availability Statement

All datasets generated for this study are included in the article/**Supplementary Material**.

## Author Contributions

X-FX, X-MQ, and Y-HJ designed this study. G-XL, CZ, and S-FY collected data. TD rechecked data. TD and X-FX performed analysis. X-FX wrote the manuscript. TD and Y-HJ reviewed the manuscript. All authors contributed to manuscript revision, and read and approved the submitted version.

## Funding

This work was supported by the National Key Research and Development Plan of China (2016YFC0106300) and Technical Innovation Major Program of Hubei province (2016ACA152), without any financial interest or benefit. The Entrusted Project of National Center for Medical Service Administration, National Health and Family Planning Commission China (No. [2019]099).

## Conflict of Interest

The authors declare that the research was conducted in the absence of any commercial or financial relationships that could be construed as a potential conflict of interest.
