# Supplementary material for: α1-Blockers and 5α-Reductase Inhibitors Are the Most Recommended Drugs in Treating Benign Prostatic Hyperplasia: An Evidence-Based Evaluation of Clinical Practice Guidelines
Source: Front Pharmacol. 2020 Mar 25;11:311. doi: 10.3389/fphar.2020.00311 (PMC7109311; doi:10.3389/fphar.2020.00311)
Supplement: Supplementary file 1 [file Table_1.docx]

Supplementary Table **1** items of AGREE **Ⅱ** tool evaluation

| **Domain** | **Item content** | **Number of items** | **Minimum score** | **Highest score** |
| --- | --- | --- | --- | --- |
| **1 Scope and purpose** | 1.The overall objective(s) of the guideline is (are) specifically described. | 3 | 3 | **21** |
|  | 2.The health question(s) covered by the guideline is (are) specifically described. |  |  |  |
|  | 3.The population (patients, public, etc.) to whom the guideline is meant to apply is specifically described. |  |  |  |
| **2 Stakeholder involvement** | 4.The guideline development group includes individuals from all relevant professional groups. | **3** | **3** | **21** |
|  | 5.The views and preferences of the target population (patients, public, etc.) have been sought. |  |  |  |
|  | 6.The target users of the guideline are clearly defined. |  |  |  |
| **3 Rigour of Development** | 7.Systematic methods were used to search for evidence. | 8 | 8 | 56 |
|  | 8.The criteria for selecting the evidence are clearly described. |  |  |  |
|  | 9.The strengths and limitations of the body of evidence are clearly described. |  |  |  |
|  | 10. The methods for formulating the recommendations are clearly described. |  |  |  |
|  | 11. The health benefits, side effects, and risks have been considered in formulating the recommendations. |  |  |  |
|  | 12. There is an explicit link between the recommendations and the supporting evidence. |  |  |  |
|  | 13. The guideline has been externally reviewed by experts prior to its publication. |  |  |  |
|  | 14. A procedure for updating the guideline is provided. |  |  |  |
| **4 Clarity of presentation** | 15. The recommendations are specific and unambiguous. | 3 | 3 | 21 |
|  | 16. The different options for management of the condition or health issue are clearly presented. |  |  |  |
|  | 17. Key recommendations are easily identifiable. |  |  |  |
| **5 Applicability** | 18. The guideline describes facilitators and barriers to its application. | 4 | 4 | 28 |
|  | 19. The guideline provides advice and/or tools on how the recommendations can be put into practice. |  |  |  |
|  | 20. The potential resource implications of applying the recommendations have been considered. |  |  |  |
|  | 21. The guideline presents monitoring and/or auditing criteria. |  |  |  |
| **6 Editorial independence** | 22. The views of the funding body have not influenced the content of the guideline. | 2 | 2 | 14 |
|  | 23. Competing interests of guideline development group members have been recorded and addressed. |  |  |  |
